# COVID-19 and a shifted perspective on infectious farm animal disease research

**DOI:** 10.1007/s10460-020-10072-2

**Published:** 2020-05-12

**Authors:** Lewis Holloway

**Affiliations:** Department of Geography, Geology and Environment, University of Hull, Hull, UK

The lockdown response to the upsurge of COVID-19 cases in the UK in March 2020 brought an immediate end to my current research project’s on-farm, in-depth social scientific fieldwork in the North of England. This research, funded by the Welcome Trust,^1^ is itself focused on persistent, endemic infectious diseases—but in our case in cattle populations. As a team of social scientists, historians, and economic and epidemiological modellers, we explore the history of Bovine Viral Diarrhoea (BVD) in the UK, examine how farmers, vets and other professionals attempt to deal with BVD in the present, and attempt to model infection patterns and how farmer behaviours affect the transmission and prevention of this disease. BVD can be transmitted between animals or passed from a cow to her calf in utero. Its effects vary in severity but it is linked to reduced productivity, various symptoms of ill-health, and increased susceptibility to other illnesses.

As a social scientist on the team, my thinking on BVD is influenced by discussions of biosecurity, or ‘making life safe’ (Bingham et al. 2008, p. 1528), a process involving anticipating what threats to life might occur, being prepared to respond to their occurrence, and being ready to make interventions to reduce the effects. Discussion of biosecurity (e.g. Hinchliffe et al. 2016) has described three overlapping ways of attempting to make life safe, and although these were originally conceived in relation to protecting of *human* life, they, and the concept of biosecurity, have more recently tended to be associated with attempts to secure *animal* life. They are, first, *exclusion* (preventing the ill moving into a space); second, *inclusion* (quarantining the ill within a space); and third, *normalisation* (managing a disease through interventions such as vaccination).

As COVID-19 took hold, we have very rapidly seen the application of all of these modes of biosecurity, which I had been thinking about in rather abstract terms and in relation to animals, back onto our own lives in very significant and concrete ways, forcing a recalibration of my perspective on animal and human infectious diseases together. We see the exclusion, inclusion and normalisation practiced by farmers in relation to their animals, being practiced by governments in relation to us through border closures, ‘social distancing’, quarantine, self-isolation, and in debates and research surrounding treatments, testing regimes, vaccination, ‘herd-immunity’ etc. Our research has been focusing on an animal disease which we have been told (e.g. by vets) should be relatively easy to eradicate through testing and/or vaccination—but BVD hasn’t been eradicated, it persists. We ask why does it persist if it’s so easy to control? COVID-19, on a different scale and rapacity as far as humans are concerned, opens up those same questions of why these things are so hard to deal with in practice, because of the complexity of viral infections and their relationships with vulnerable bodies, the logistics of organising medical equipment and care, and the messiness and recalcitrance of human behaviour in relation to ‘lockdown’ regimes. Exemplifying this human-animal parallel, *mobility* is crucial in thinking about infection. For an agricultural and food system to function people and animals *must* move, in ‘normal’ circumstances and even in lockdown, in order for food production to continue, but at the same time movement and the mingling of human and animal bodies facilitates infection. In lockdown, too, addressing viral diseases in animals may be even harder as animal biosecurity and care become more challenging because of attempts to manage human biosecurity, let alone due to human illness affecting farm work.

While glib notions of ‘One Health’ (emphasising the interconnectedness of human and animal health) might be criticised for their lack of specificity to particular situations,^2^ there is still a sense in which these parallels and interconnections suddenly become more graphic as we see them playing out in our lives in relation to something clearly much more infectious and problematic for us as humans, something shocking our healthcare, social care and political-economic systems. There is a sudden brutal exposure of the entanglement of human and animal lives, not just in terms of the presumed origins of COVID-19 in ‘wild’ animals and their use as food in certain parts of the world, but in a more mundane way in terms of a sense of shared embodiedness, vulnerability, and subjection to similar biosecurity measures. Our food systems and lifestyles together produce these disease risks—in livestock farming systems, and in mobile and interconnected human lives: both provide ideal viral environments.

## Footnotes

1 Wellcome Trust Collaborative Award 209,818/B/17/Z. Farm Level Interdisciplinary Approaches to Endemic Livestock Disease https://field-wt.co.uk/

2 Thank you to Abigail Woods for her advice on ‘One Health’ concepts.
